# Deciphering Lung Adenocarcinoma Heterogeneity: An Overview of Pathological and Clinical Features of Rare Subtypes

**DOI:** 10.3390/life13061291

**Published:** 2023-05-31

**Authors:** Andrea Mogavero, Paolo Bironzo, Luisella Righi, Alessandra Merlini, Federica Benso, Silvia Novello, Francesco Passiglia

**Affiliations:** Department of Oncology, University of Turin, San Luigi Gonzaga Hospital, 10043 Orbassano, Italy; mogavero.andrea@gmail.com (A.M.);

**Keywords:** pulmonary enteric carcinoma, colorectal cancer, immunochemistry, mutation analyses, colloid carcinoma, fetal carcinoma, NOS carcinoma

## Abstract

Lung cancer is one of the most frequently diagnosed cancers worldwide and the leading cause of cancer-related death. The 2021 World Health Organization (WHO) classification provided a detailed and updated categorization of lung adenocarcinomas with a special focus on rare histological types, including enteric, fetal and colloid types, as well as not otherwise specified adenocarcinoma, overall accounting for about 5–10% of all cases. However, rare entities are nowadays difficult to diagnose in most centers, and evidence of optimal therapeutic management for these patients is still lacking. In recent years, increasing knowledge about the mutational profile of lung cancer, in addition to the spreading diffusion of next-generation sequencing (NGS) in different centers, have been helpful in the identification of rare variants of lung cancer. Hence, the hope is that several new drugs will be available in the near future to treat these rare lung tumors, such as in targeted therapy and immunotherapy, which are often used in clinical practice for several malignancies. The aim of this review is to summarize the current knowledge about the molecular pathology and clinical management of the most common rare adenocarcinoma subtypes in order to provide a concise and updated report that can drive clinicians’ choices in their routine practice.

## 1. Introduction

Lung cancer is one of the most frequently diagnosed cancers worldwide and the leading cause of cancer-related death. The worldwide lung cancer mortality rate amounted to 1.59 million deaths in 2012, accounting for 19.4% of the total cancer deaths, while in terms of incidence, it is second only to prostate and breast cancers among men and women, respectively [1].

Over the past decade, a significant reduction in lung cancer mortality has been observed for both sexes in the United States [2], mostly driven by the introduction of lung cancer screening and precision medicine in clinical practice [3]. Conversely, lung cancer-related mortality is rising in the female European population, requiring different approaches in terms of both diagnostic and therapeutic interventions [4].

**Figure 1:**
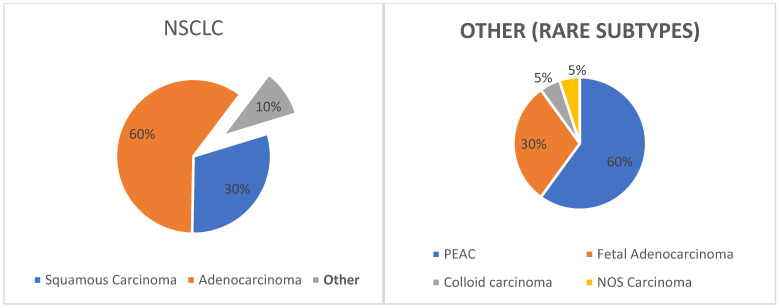
NSCLC and classification of rare variants based on World Health Organization (WHO) 2021 classification. PEAC: pulmonary enteric adenocarcinoma; NOS: not otherwise specified.

Based on morphological features, lung cancer has been historically classified as small cell lung cancer (SCLC—15%) and non-small cell lung cancer (NSCLC—75%), the latter including adenocarcinoma (ADC—60%), squamous cell carcinoma (Sq—30%) and other less common subtypes (10%) (Figure 1).

A sex-specific difference in the incidence of lung cancer subtypes has been reported, with women most likely to develop adenocarcinoma rather than squamous cell carcinoma, which is most commonly detected in men [5]. Even if tobacco smoking remains the main risk factor, being involved in the development of almost 70% of lung cancer cases, the increasing proportion of non-small cell lung cancer diagnoses in people who have never smoked has led to the hypothesis that this subgroup of tumors should be considered a separate entity [6] with specific molecular and genetic features, since they are typically found in younger female patients harboring oncogenic driver alterations, especially the adenocarcinoma subtype. Other environmental risk factors include air pollution, occupational exposure (asbestosis, metals, etc.) and chronic obstructive pulmonary disease [7]. 

The recent introduction of innovative therapies (e.g., targeted therapies and immunotherapy) in clinical practice has certainly changed the therapeutic management of lung cancer patients, but only a limited proportion of them currently experience long-term clinical benefits in real-world scenarios, while the 5-year survival rate in the overall lung cancer population remains poor, especially for patients affected by SCLC and other rare histological subtypes, which exhibit relevant differences in terms of epidemiology, molecular characteristics and prognosis [8]. 

In recent years, both improvements in molecular techniques, such as next-generation sequencing (NGS), and immunohistochemistry have led to a better classification of lung adenocarcinoma subtypes and the biological mechanisms underlying these entities. The 2015 WHO classification introduced many important changes largely owing to the remarkable progress in understanding genetics and molecular-targeted therapies [9]. The more recent 2021 World Health Organization (WHO) classification provided a detailed and updated categorization of lung adenocarcinomas with a special focus on rare histological types, including the enteric, fetal and colloid types, as well as not otherwise specified adenocarcinomas, overall accounting for about 5–10% of all cases. With this new classification, thanks to the introduction of new immunohistochemistry biomarkers and molecular testing, many of the more sophisticated approaches to pathologic diagnosis have led to more precise pathologic and genetic classification of lung tumors. Moreover, the understanding of the biological mechanism underlying lung cancer transformation has been improved, leading to more personalized medicine, even for rare lung cancers.

However, rare entities are still difficult to diagnose and manage in most centers because of the low incidence of these tumors, for which reliable data and prospective trials are still lacking. 

Most of the evidence is currently derived from retrospective studies employing data collection, case reports published in the literature, subgroup analyses of clinical trials or international guidelines for these rare tumors’ common counterparts.

Consequently, there is no standard of care for the management of rare variants of lung cancer, while treatment algorithms are defined by each center and international, univocal guidelines are still lacking.

For this reason, the aim of this review is to summarize the current knowledge about molecular pathology, diagnostic tools and clinical management for the most common rare adenocarcinoma subtypes, with a focus on pulmonary enteric carcinoma, fetal carcinoma, colloid carcinoma and NOS carcinoma, in order to provide a concise and updated report that can drive clinicians to better management of these rare tumors, both in localized and advanced settings.

## 2. Methods

We searched for articles about pulmonary enteric adenocarcinoma (PEAC), fetal carcinoma (FC), colloid carcinoma (CC) and NOS ADC in PubMed, including case reports, original articles and reviews. 

## 3. Pulmonary Enteric Adenocarcinoma

Pulmonary enteric adenocarcinoma (PEAC) is a rare subtype of lung adenocarcinoma that was first described in 1991 by Tsao and Fraser [10]. This entity has been included in the WHO classification since 2015, while in 2011, it was added to the IASLC/ATS/ETS classification. Only a few cases have been described worldwide [11]; in particular, in case reports with heterogeneous therapeutic management. 

PEAC is described as a primary lung adenocarcinoma that contains more than 50% enteric differentiation with typical morphology and immunochemistry staining for at least one marker of colorectal cancer (CRC), such as cytokeratin-20 (CK-20), caudal-related homeodomain protein 2 (CDX-2) or mucin 2 (MUC2). Due to the different treatment strategies and prognoses, it is important to distinguish PEAC from metastases of colorectal cancer (MRC), which can have similar morphological and immunohistochemical patterns. The clinical presentation is similar to that of conventional lung ADC (CLA) and the differential diagnosis can be difficult, requiring imaging, histopathology and immunochemistry analyses. The treatment strategy is similar to that of primary lung adenocarcinoma, including surgery, radiotherapy and systemic therapies depending on the clinical stage. In recent years, the significant advances in molecular pathology have made PEAC diagnoses more widely accessible. Due to its rarity, most of the evidence derives from case reports and retrospective series, while prospective studies are absent. 

### 3.1. Epidemiology

The overall PEAC prevalence accounts for 0.5% of all NSCLC cases [12,13]. The age of presentation is generally between 50 and 60 years and patients usually present more advanced disease at diagnosis compared to those affected by CLA, although some cases with early-stage disease have been described [14]. Zhao et al. showed that PEACs are more common in male adult patients [12,15,16], but epidemiological data remain controversial because of the limited records reported in the literature. 

The etiology of PEAC is not completely understood. The first hypothesis, as put forward by Satoh et al. [17], is based on the presence of common cancer stem cells in the lower respiratory tract and gastrointestinal mucosa, including goblet cells, Paneth cells and neuroendocrine cells. The role of smoking is still controversial [15,17], with some studies showing no differences in terms of smoking status between PEAC and primary lung adenocarcinoma [12,13]. 

### 3.2. Clinical Characteristics

The clinical presentation of PEAC is similar to that of CLA, with the most common symptoms being dry cough, dyspnea, fever, hemoptysis and chest pain, even though some patients can be completely asymptomatic [12,18]. Radiology examinations usually show nodular lesions with associated pleural effusion; interestingly, ground glass opacity (GGO) has not been described as a radiological presentation in PEAC patients [12]. Compared to other lung cancer subtypes, PEAC patients have larger lesions characterized by satellite nodes being confined to the peripheral rather than central fields [12]. Retrospective data suggest that PEAC may usually be found in the right lung and only rarely in the left lobes [10,16], and recent retrospective analyses have shown that idiopathic pulmonary fibrosis (IPF) could be the primary radiological presentation of PEAC [19]. PEAC is an aggressive tumor, often spreading outside of the thorax, including to the bone, liver, lymph nodes and pleura [16,20], the latter being the most frequent site of metastases. However, even rare metastatic sites, including the skin and pancreas, have been described [21,22].

Despite these data, there are only few radiological differences between PEAC and lung metastases from colorectal cancer (MRC). Therefore, colonoscopy and gastroscopy should always be recommended for patients with suspected PEAC in order to rule out a primarily gastrointestinal cancer origin. 

A retrospective study including 18 PEAC cases showed that some serum blood biomarkers could be identified, even though their sensitivity and specificity were low. Specifically, carcinoembryonic antigen (CEA) and carbohydrate antigen (CA 19.9) levels were significantly higher than those for the cytokeratin-19 fragment (CYFRA 21-1) and neuron-specific enolase (NSE) [23,24]. Moreover, PEAC patients seem to have higher levels of both CEA and CA 19.9 compared to patients affected by CLA [23]. 

The prognosis of PEAC remains poor, although data are still scant. In a retrospective study, Whang et al. collected clinicopathological characteristics for nine cases of PEAC. Eight of patients died after a brief follow-up of 2–60 months due to disease progression, with an average period of time of 23.9 months [16]. 

### 3.3. Morphology and Immunochemistry

PEAC shares its morphological and immunochemistry patterns with primary lung adenocarcinoma and colon–rectal cancer [23]; thus, due to the differing treatment management, differential diagnosis should be undertaken accurately. 

**Figure 2:**
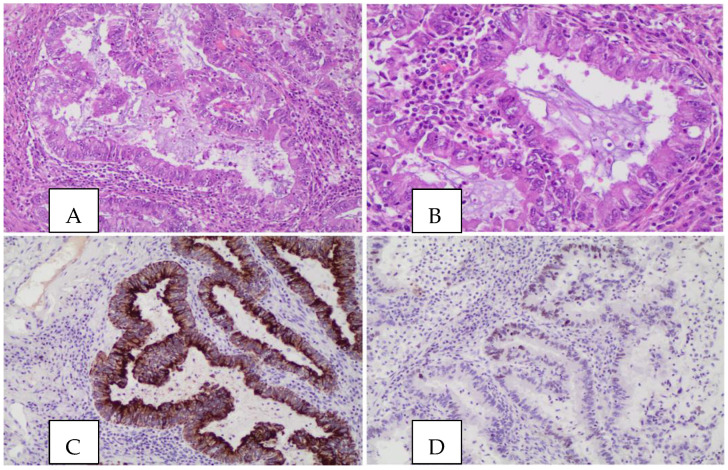
Histological and immunohistochemical pictures of PEAC: (**A**) glandular architecture lined by tall columnar cells with an eosinophilic cytoplasm and enteric appearance (EE 10×); (**B**) higher magnification (EE 20×); (**C**) CK7 (10×) shows diffuse positivity, while TTF1 (**D**) is only focally positive (10×).

Based on the 2021 WHO classification, PEAC is defined as a lung adenocarcinoma with enteric differentiation (>50%) that is mainly characterized by tall columnar cells with an eosinophilic cytoplasm that can display an acinar, cribriform or papillary architecture [13,23] (Figure 2). Some glands may contain necrotic material defined as “Dirty Necrosis”. 

Regarding morphological aspects, PEAC cells have brush borders and prominent nucleoli; the polarity of the nuclei is normally preserved, even though some cells with low differentiation can lose it. These tumors may have different grades of differentiation. Poorly differentiated tumors show solid growth patterns, while well-differentiated tumors usually have a gland pattern and moderately differentiated ones have a cribriform pattern. 

Inflammatory infiltrate can be found around tumor cells with an abundant matrix and different subtypes of interstitial cells, such as goblet and Paneth cells with neuroendocrine differentiation [16]. 

As the differential diagnosis of PEAC and colon–rectal and lung cancer metastases (MCR) can be quite difficult, a panel of immunochemistry tests should be used. Specifically, to confirm PEAC diagnosis, at least one lung and one enteric biomarker must be simultaneously expressed. 

Thyroid transcription factor 1 (TTF-1), cytokeratin 7 (CK-7) and napsin-A (an aspartic protease expressed by the cell surface of type II pneumocytes) are the most common immunohistochemistry assay targets used to identify a lung origin, while CDX-2, CK-20 and villin can be used to identify an enteric origin [16]. CDX-2 is usually expressed in 75–80% of cases; the lung markers CK-7 and napsin-A are expressed in 90% and 84% of cases, respectively [25]; TTF-1 shows low expression, although the sensitivity and specificity evidence is poor [13,23]. SATB-2 and beta-catenin have been recently discovered as further potential markers [15]. Notwithstanding the fact that this diagnostic model is often heterogeneous, Zhang et al. highlighted that the immunochemistry patterns of PEAC mainly resemble lung cancer due to the expression of CK-7 and TTF-1, despite CDX-2 and CK-20 being typically expressed in CRC and MRC [26].

Furthermore, immunohistochemistry staining has diagnostic value only in a few cases. For this reason, there is an urgent need to identify new reliable biomarkers for the accurate differential diagnosis of PEAC and MCR. 

Chen et al. retrospectively analyzed 129 cases of PEAC and 50 cases of colorectal cancer (CRC), suggesting that the combined positive expression of CK-7 and CDX-2 markers provides more specificity and sensitivity for differential diagnosis [23,24]. A combination of villin and CDX-2 can also be used to differentiate PEAC from MRC. If both markers are negative, a gastrointestinal origin can be excluded.

Even if differentiating PEAC and MRC is often difficult due to the absence of specific biomarkers, the advent of NGS has enhanced knowledge about the molecular patterns of lung cancer, leading to improved differential diagnosis using biomarker identification.

Zhang et al. revealed that PEAC molecular profiles resemble lung cancer, including ERBB2, EGFR and ALK mutations accounting for 75% of cases; on the other hand, MRC and CRC involve alterations of adenomatous polyposis coli (APC) genes, the KRAS pathway and mutation of mismatch repair genes (MSH6, PMS, MLH1). No classic CRC mutations have been detected in PEAC. The authors also analyzed the mutational load of these entities and showed that CRC has the highest among them, including primary missense mutations, nucleotide substitutions, copy number variations and loss of function [27]. 

Furthermore, Zhao et al. performed whole-exome sequencing (WES) with a cohort of 32 PEAC patients to identify genetic and epigenetic alterations that could improve the diagnostic differentiation from MRC. APC mutations could be a major biomarker for distinguishing MRC from PEAC, although they are also found in approximately 6% of lung cancer [28]. 

Zuo et al. underlined that aberrant methylation of the HOX clusters of genes, including HOXA9 and HOXA4, involved in oncogenesis and hypomethylation of GCNT2, was closely related to lymph node metastases in PEAC [28]. This new oncological frontier based on epigenetic and molecular investigation could be helpful to consolidate the diagnosis of rare variants of lung cancer. 

Finally, it is important to note that, in recent years, some chromosomal aberrations have been discovered. Focusing on rare malignancies, the most frequent chromosome alterations in PEAC are loss of chromosomes 3p and 1q and gain of chromosome 1p [24]. 

Concerning differential diagnosis, it is important to mention invasive mucinous adenocarcinoma (IMA), accounting for 3% to 10% of ADCs, which should be accurately distinguished from PEAC. It usually presents with multifocal, multilobar and bilateral disease. Based on the 2021 WHO classification, this entity usually shows a lepidic growth pattern and has a goblet-cell morphology [29]. Immunohistochemistry is often characterized by CK7+, HNF4+ and TTF-1 and focal positive expression of CDX-2 and CK20 biomarkers [30].

### 3.4. Molecular Pathology

Genetic mutations play an important role in the pathogenesis of lung cancer. Owing to its rarity, the role of molecular alterations in PEAC is controversial, although recent evidence from retrospective studies and case series has been reported.

Kirsten rat sarcoma viral oncogene homologue (KRAS) is the most frequently altered gene in PEAC, occurring in 40–60% of cases [13,16,24], while classical epidermal growth factor receptor (*EGFR*) mutations (L858R mutation and exon 19 deletion) have been reported less frequently. The p.G12V variant is the most common KRAS mutation [24] and is probably associated with negative expression of CK-7 [12]. 

Interestingly, human epidermal growth factor receptor-2 (ERBB2) alterations were found in 44% of PEAC cases, especially gene amplifications and insertion mutations [10,23]. Conversely, anaplastic lymphoma kinase (ALK) rearrangements and *BRAF* mutations have rarely been reported [10], while no cases of *MET* or *ROS-1* alterations have yet been described [24]. Todisco et al. described point mutation of both SMAD4 and FLT3 genes in a cutaneous metastasis of PEAC responsible for the production of a truncated protein implicated in signal transduction cell processes, such as proliferation, survival and differentiation [21]. 

Programmed death ligand-1 (PD-L1) seems to be highly expressed (with a cut-off of major than 50%) in more than 50% of patients with PEAC [13]. Moreover, mismatch repair (MMR) gene products, such as MSH6, MSH2, MLH1 and PMS2, are sometimes altered in PEAC [15], leading to genomic instability. 

Finally, Chen et al. described a higher molecular tumor burden (TMB) in PEAC compared to CLA, although evidence is still lacking [23]. 

### 3.5. Treatment Strategies

The treatment strategies available for PEAC therapy are similar to those reported for lung adenocarcinoma and include chemotherapy, target therapy, surgery and radiotherapy. Surgical resection is the primary choice for localized disease, while locally advanced malignancies can be treated with sequential or concomitant chemo-radiotherapy. Finally, inoperable and metastatic disease should be treated exclusively with standard chemotherapy based on platinum doublets and pemetrexed; chemotherapy regimens for CRC had no significant effect on PEAC [10]. 

Immunotherapy is considered a major breakthrough in cancer treatment, with routine clinical use for melanoma, renal cancer and lung cancer. PD-1/PD-1 inhibitors (*pembrolizumab*, *cemiplimab*, *atezolizumab*) have shown better results in terms of overall survival (OS) and progression-free survival (PFS) than standard platinum based-chemotherapy in patients with PD-L1 ≥ 50%. At the same time, immunotherapy can be efficaciously associated with standard chemotherapy based on platinum doublets according to international guidelines [31]. 

Chen et al. stated that PEAC could show a higher TMB than lung adenocarcinoma, thus further increasing the likelihood of response to immunotherapy [23]. However, Chun et al. described a case report demonstrating hyper-progression after one cycle of immunotherapy in one patient affected by metastatic PEAC harboring KRAS mutations [26]. Finally, a recent retrospective collection of 10 patients affected by PEAC demonstrated low numbers of CD8+ T cell tumor-infiltrating lymphocytes in the tumor microenvironment, associated with a low TMB and low expression of PD-L1, compared to CLA [14]; for this reason, the role of immunotherapy in PEAC is still controversial. 

## 4. Fetal Adenocarcinoma

Fetal lung adenocarcinoma is a very rare cancer described in 1982 as a subtype of pulmonary blastoma. Well-differentiated fetal adenocarcinoma (WDFA) accounts for 0.5% of all lung neoplasms [32]; it is more commonly detected in young female smokers and is usually associated with a good prognosis [33]. Reports describing the radiological features of this disease are limited, but it is usually characterized by round-shaped, well-defined solid lesions with oval or lobulated borders and high FDG-PET uptake [34]. From the histological point of view, WDFA is characterized by neoplastic glands and tubules lined with non-ciliated columnar cells, with some of them resembling endometrial glands and squamous morula cells with a fibroblastic stroma [34,35]. Rounded cells with eosinophilic cytoplasm are frequently observed [34]. These glands are rich in glycogen, as demonstrated by periodic acid–Schiff (PAS) reaction staining. Immunohistochemistry is usually positive for epithelial markers associated with vimentin and synaptophysin [35]; beta-catenin and WNT signaling pathway abnormalities are crucial in the pathogenesis of WDFA, with upregulation of c-myc and cyclin-D1 frequently reported in these tumors. Moreover, diagnosis can be challenging using only small biopsy specimens, and anatomical resection with surgical specimens should be preferentially considered. Differential diagnosis must be considered with regard to pulmonary blastoma—which is a biphasic tumor with a sarcomatous and carcinomatous component characterized by aggressive behavior—fibrous solitary tumors, Castleman’s disease, inflammatory pseudotumors, lymphoma and sarcoidosis. WDFAs are usually smaller than 5 cm and rarely spread to lymph nodes or the pleural surface. For this reason, surgical approaches are usually preferred and associated with good survival outcomes [36]. 

Indeed, following surgical resection, 5-year and 10-year survival rates have been reported to be around 80% and 75%, respectively. [36]. Combined treatment with radiotherapy and chemotherapy (based on platinum doublets) should be considered for patients with locally advanced disease, while metastatic disease can be treated with chemotherapy alone, although prognosis remains poor [36]. 

In contrast, high-grade fetal adenocarcinoma (HGFA) is a poorly differentiated variant, less frequently diagnosed compared to WDFA and predominantly occurring in male patients [34]. HGFA is characterized by disorganized glands with large vesicular nuclei, prominent nucleoli, anisonucleosis and adenocarcinoma foci [35]. Morula differentiation is absent, and positive stains for p53 and alpha-fetoprotein (AFP) are evident. Treatment consists of systemic chemotherapy, although responses are rare, so prognosis remains poor. 

## 5. Colloid Adenocarcinoma 

Colloid adenocarcinoma (CA) of the lung was first described in 1992 and identifies a group of very rare lung tumors characterized by abundant extracellular mucin associated with scant neoplastic epithelium, distorting the alveolar space [37]. In recent years, these tumors have been reported under different designations, including “Mucinous Cystadenoma” and “Mucinous Cystic Adenocarcinoma”. Recently, this entity was included in the 2021 WHO classification as a rare subtype. Although these tumors can show indolent biological behavior, they sometimes spread to distant sites, including lymph nodes, bone and brain [38]. CA occurs more frequently in females and smokers, with a median age at diagnosis of 65 years [39]. 

Macroscopically, CA usually presents as a large lesion predominantly composed of gelatinous/mucinous material. Microscopically, it is characterized by an abundant mucin matrix with some tumor cells floating in the mucous, including both cuboidal and columnar cells, with small nuclei and without atypia or mitotic activity [38]; fragments of alveolar walls are frequently noted.

Regarding immunohistochemistry features, research on colloid cancer has shown some overlapping findings with PEAC, including strong expression of CK7 and moderate expression of CK20, CDX-2 and MUC2 [39]. TTF-1 and napsin-A are normally negative, while weak expression of surfactant protein-A (SP-A) can be present [38]. However, some CAs may show positive TTF-1 staining due to tumor heterogeneity [39]. Some reports suggest that *KRAS* mutations on codon 12 or 13 could be found in CA [38]. 

This entity usually presents as a peripheral and solitary nodular intrapulmonary mass, which may be discovered incidentally during a routine radiographic evaluation [39]. The main treatment is surgery; although most patients have favorable outcomes, some of them develop recurrences, especially when associated with a major non-colloid part or KRAS mutations. Although there are no established diagnostic criteria, this designation is used for tumors that are composed exclusively of colloid cancer or have only small foci of non-colloidal tissue [39]. 

## 6. NOS Carcinoma

Not otherwise specified lung carcinoma (NOS carcinoma) is a rare, usually aggressive, variant of NSCLC [40]. It usually presents as locally advanced or metastatic disease and more frequently occurs in males, with a median age of 70 years at diagnosis [40]. 

Diagnosis is often challenging because of the scarcity of specimens and the highly heterogeneous composition of these tumors, which limit the accuracy of subtyping biopsies [41]. Furthermore, NOS subtypes may be assigned to other subcategories of malignancy, such as pleomorphic or large cell tumors, due to their poor differentiation [41]. 

For more accurate pathological diagnoses, immunochemistry panels could be helpful [42]. Based on several markers and immunochemistry staining, NOS carcinoma can be divided into two subcategories: (1) NSCLC favoring ADC, occurring in 40% of cases and usually expressing both TTF-1 and napsin-A; and (2) NSCLC favoring squamous tumors, occurring in 10% of cases and characterized by p40-positive stains. Only when these markers are both negative can a diagnosis of conventional NOS carcinoma be confirmed. 

Due to its rarity and difficulties in diagnosis, molecular pathology identification is quite challenging. EGFR mutations and ALK rearrangements are the most common molecular alterations more frequently detected in NSCLCs favoring ADC and squamous tumors than conventional NOS carcinoma [43]. 

NOS carcinoma usually has an aggressive tumor biology with a poor prognosis. Systemic chemotherapy with platinum doublets is the best treatment, although surgery should be evaluated for localized disease, while chemo-radiotherapy may be effective in locally advanced cases. 

## 7. Conclusions

The 2021 WHO classification recently introduced a special focus on the rare subtypes of lung cancer and highlighted that, in the definition of tumor morphology, molecular detail should be always implemented. 

Rare tumors are characterized by great heterogeneity in terms of diagnosis, treatment strategy and prognosis (Table 1). Furthermore, accurate diagnosis is challenging because of the limitations of diagnostic assays, and a surgical specimen is still usually essential for a detailed pathological assessment. Surgery is the cornerstone of treatment for localized disease, while cytotoxic chemotherapy represents the standard of care for advanced disease, and the potential efficacy of targeted therapies and immunotherapy against these rare subtypes remains to be proven. For these patients, a diagnostic and therapeutic workup should be carried out in a reference center and shared with other physicians, such as thoracic surgeons, radiotherapists, radiologists and oncologists, in the context of multidisciplinary team discussions in order to optimize the diagnostic and therapeutic management of this disease.

In the upcoming years, due to the widespread diffusion of new diagnostic technologies and tools, such as next-generation-sequencing techniques and liquid biopsy, our knowledge about the molecular basis of these entities is expected to increase, supporting the design of higher numbers of prospective trials with novel drugs and combinations that can be expected to further improve the 5-year survival rate in these patients. 

Moreover, the application of new diagnostic tools, such as epigenetic and tumor microenvironment analyses and TMB, will be helpful in defining more accurate treatment strategies, making it possible to also implement a personalized medicine approach in the context of rare lung cancer subtypes. 

## Figures and Tables

**Table 1:** Clinicopathological characteristics of rare subtypes of lung adenocarcinoma.

Subtypes	Epidemiology	Gender	Smoking Status	IHC	Molecular Pathology	Treatment
PEAC	0.5%	Males > females	Smokers	CK7, CDX-2, villin	KRAS G12V, ERBB2, EGFR (del ex19 and L8S8R)	Surgery/systemic treatment **
Fetal adenocarcinoma	0.5%	Young females *	Smokers	Synaptophysin, vimentin	WNT signal	Surgery ***
Colloid carcinoma	0.1%	Females > males	Smokers	CK7, CDX-2, CK20, MUC2	KRAS codons 12 and 13	Surgery/systemic treatment
NOS carcinoma	0.1%	Males > females	Unknown	TTF-1 and p40 or none ****	EGFR and ALK-EML4	Systemic treatment

* Especially for well-differentiated fetal lung adenocarcinoma (WDFA); high-grade fetal adenocarcinomas are more frequent in males. ** Surgery is feasible for localized disease. Systemic treatments include chemotherapy and radiotherapy. *** High-grade fetal adenocarcinomas are treated with systemic therapy. **** TTF-1 is especially expressed in NOS adenocarcinoma variants, while p40 is expressed in NOS squamous variants. Some of the NOS carcinomas do not express any biomarkers.

## Data Availability

Not applicable.
